# Cautions on utilizing plasma GFAP level as a biomarker for reactive astrocytes in neurodegenerative diseases

**DOI:** 10.1186/s13024-025-00846-9

**Published:** 2025-05-09

**Authors:** Wongu Youn, Mijin Yun, C. Justin Lee, Michael Schöll

**Affiliations:** 1 Center for Cognition and Sociality, Institute for Basic Science, Daejeon, 34126 Republic of Korea; 2 Department of Nuclear Medicine, Yonsei University College of Medicine, Seoul, 03722 Republic of Korea; 3 Department of Psychiatry and Neurochemistry and the Wallenberg Centre for Molecular and Translational Medicine, University of Gothenburg, Gothenburg, 41345 Sweden; 4 Dementia Research Centre, Queen Square Institute of Neurology, University College London, London, WC1E UK; 5 Department of Neuropsychiatry, Sahlgrenska University Hospital, Mölndal, 43141 Sweden

**Keywords:** Biomarker, Glial fibrillary acidic protein, Neurodegenerative disease, Reactive astrocyte

## Main text

In the recent decade, there has been a surge of efforts to develop scalable, specific and cost-effective biomarkers in blood to diagnose neurodegenerative diseases and prognose their progress even before overt symptoms manifest. Among an array of brain-associated proteins, glial fibrillary acidic protein (GFAP) has emerged as a compelling biomarker candidate, often in conjunction with other biomarkers. GFAP levels in bodily fluid, especially blood and cerebrospinal fluid (CSF), have underscored associations with disease progression by robust support in a substantial body of reports encompassing cohorts afflicted with a spectrum of brain and spinal cord disorders, including progressive neurodegenerative diseases such as Alzheimer’s disease (AD), Parkinson’s disease, multiple sclerosis and Lewy body dementia. Notably, GFAP in CSF is known to reflect astrogliosis in alignment with other astrogliosis marker levels such as S100β, chitinase-3-like protein 1 (CHI3L1, also known as YKL40 in humans and BMP39 in mice), aquaporin 4, evidence in tissue by immunohistochemistry staining, and uptake of certain PET radiotracers targeting reactive astrocytes, i.e., ^11^C-deuterium-L-deprenyl (^11^C-DED), ^11^C-BU99008, ^11^C-SMBT-1 or ^11^ C-acetate [1]. On the other hand, GFAP levels in blood seem to demonstrate more precise diagnostic performance than CSF GFAP level in an AD context. Patient case studies employing MRI and PET have underscored correlations between disease progression and GFAP levels in bodily fluids, with plasma GFAP yielding greater significance [2]. Furthermore, recent cohort studies suggest that the effect of amyloid-β (Aβ) on tau pathology may be modulated by astrocytic reactivity, which was suggested to be indicated by increased plasma GFAP levels [3]. The recent inclusion of interchangeable use of plasma and CSF GFAP as a marker of inflammation (category ‘I’) in the Alzheimer’s Association Workgroup criteria for diagnosis and staging of Alzheimer’s disease showcases its suggested diagnostic potential [4]. We argue, however, that there are several concerns regarding the use of blood GFAP as a direct biomarker for astrocyte reactivity. Research has identified discrepancies between astrocyte reactivity examined by ^11^C-deuterium-L-deprenyl (^11^C-DED) PET imaging and plasma GFAP levels in AD patients [5], with more significant changes observed in blood GFAP levels than in cerebrospinal fluid (CSF) GFAP levels [6]. In this perspective, we argue that astrocytic reactivity cannot be represented solely from blood GFAP level, and more direct methods for examining astrocyte reactivity such as PET imaging must be followed. Our argument is based on two primary concerns: the ambiguous origin of plasma GFAP and inconsistencies between blood GFAP level increases and other biomarkers.

First, the origin of blood GFAP remains unclear, with uncertainty about whether plasma GFAP derives from CSF or specifically from (reactive) astrocytes. Identified in multiple sclerosis brain tissue in 1969, GFAP quickly became a key astrocyte marker and has since been widely used for selectively targeting astrocyte expression in mice via the GFAP promoter [7]. During both pathological and physiological states, the well-established process of reactive astrogliosis leads to morphological and functional changes in astrocytes, accompanied by increased GFAP expression. Consequently, elevated GFAP levels in CSF and blood have traditionally been attributed to upregulated production by reactive astrocytes. While GFAP release into the bloodstream is well-documented in acute brain injuries with transient blood-brain barrier disruptions, the mechanisms behind increased blood GFAP levels in neurodegenerative disease progression—especially when blood-brain barrier integrity is maintained—remain elusive.

Recent studies further question this assumption, revealing a negative correlation between plasma GFAP levels and astrocytic reactivity in both autosomal dominant and sporadic AD cases, challenging earlier assumptions [5]. Also, a recent preprint reported a negative correlation between brain and plasma GFAP concentration in a 5xFAD transgenic mouse model [8]. Notably, in AD, glial activation appears to precede increases in both CSF and plasma GFAP levels, suggesting that elevated plasma GFAP may not solely originate from GFAP-upregulated reactive astrocytes [1]. During the pre-symptomatic phase, modest increases in CSF and plasma GFAP are observed even 10 years before symptoms manifest [8], with more significant elevations arising only in symptomatic stages. Additionally, plasma GFAP levels tend to increase before CSF GFAP, with differences in the magnitude of these increases [6]. In contrast, increased serum GFAP levels have shown correlations with immunohistochemistry-based astrocytic reactivity and post-mortem brain atrophy in dementia patient cohort study [9]. This temporal and spatial discrepancy calls into question the direct association of blood GFAP with astrocytic reactivity.

The discrepancy between CSF and plasma GFAP levels is not the only point of doubt; GFAP expression in various body cells also raises questions about the true origin of blood GFAP. Although GFAP is widely regarded as an astrocyte-specific protein, its roles remain poorly understood, partly due to its variable expression across different brain cell types and astrocytic subpopulations. Even within the human brain, additional GFAP-expressing cells, such as developing neural progenitor cells and ependymal cells, are present, requiring supplementary markers like calcium-binding protein B (S100β), excitatory amino acid transporter 1 (EAAT1 or GLAST), glutamine synthetase (GS), and aldehyde dehydrogenase 1 family member L1 (ALDH1L1) for accurate astrocyte identification. Beyond the central nervous system, GFAP expression is also found in non-myelinating Schwann cells in the peripheral nervous system (PNS), Müller glia in retina, enteric glial cells in the enteric nervous system (ENS), renal tubular cells, Leydig and Sertoli cells in the testis, and various cell types in the liver, skin, bone, and placenta under normal conditions [7].

Notably, these non-brain GFAP-expressing cells in pathological conditions also upregulate GFAP, complicating attempts to pinpoint the origin of blood GFAP. For instance, GFAP is overexpressed in the intestines of patients with inflammatory bowel disease; Parkinson’s disease has been associated with elevated GFAP expression and phosphorylation in enteric glia; hepatic stellate cells show GFAP overexpression near areas of hepatic fibrosis; and GFAP has been detected in the bloodstream following complex thoracic aortic surgery [10]. Despite these observations, no direct evidence yet demonstrates that blood GFAP originates from reactive astrocytes in the brain.

Second, inconsistencies are documented for the association between plasma GFAP levels and other glial biomarkers, whereas CSF GFAP levels are strongly correlated with these markers. Glial biomarkers extend beyond GFAP alone, with different markers of reactive astrocytes, such as CHI3L1 and S100B, as well as soluble triggering receptor expressed on myeloid cells 2 (sTREM2), which is secreted by microglia. While CSF GFAP levels correlate with these glial biomarkers, contradictory reports exist regarding the correlation between plasma GFAP levels and measures of astrogliosis as investigated with different PET tracers or at autopsy. Plasma GFAP levels were thus positively correlated with ^18^F-SMBT-1 uptake in sporadic AD patients compared to control groups [11], but showed no correlation or were even negatively correlated with ^11^C-DED [5] or GFAP levels in brain tissue [8], suggesting that mechanisms beyond reactive astrocytes or CSF release may contribute to elevated blood GFAP levels [6]. Moreover, in a cohort study on multiple sclerosis (MS), serum GFAP levels failed to predict disease activity and progression, whereas CSF GFAP levels were significant predictors despite a correlation existing between CSF and serum GFAP levels and other glial/neuroinflammation markers [12].

Delving into more practical considerations, quantifying GFAP levels in blood is challenging for conventional ELISA methods, which has led to the adoption of ultrasensitive techniques such as SIMOA. However, the inconsistencies in GFAP levels across studies indicate a lack of standardized criteria for its use as a biomarker, which might be due to limitations in antibody-based methods, including the aggregation-related hook effect and the presence of multiple GFAP isoforms and post-translational modifications [13]. To advance GFAP as a more reliable biomarker, standardized quantification methods, sample handling protocols including antibody information, and comprehensive studies on GFAP isoforms are essential to clarify the origins of GFAP release and improve its analytical accuracy.

Despite the numerous limitations and unresolved issues surrounding blood GFAP level elevations, these levels remain broadly accepted as biomarkers reflecting neurodegenerative disease stages, not only specific to AD but also early amyloidosis, dementia or faster cognitive decline [4]. Alongside other markers such as phosphorylated tau, amyloid beta 42/40, and neurofilament light chain protein (NfL), blood GFAP is believed to enhance our understanding of disease progression. However, for GFAP to be recognized as a reliable biomarker, a rigorous examination of its origins and causal links to pathophysiological conditions is essential, grounded in concrete biological evidence rather than correlations alone.

To truly determine GFAP’s value, we must undertake a comprehensive investigation that includes mapping GFAP expression across all relevant tissues, selectively marking or targeting GFAP in specific cell types, for example, along with PET imaging targeting particular cells, and closely examining the conditions that trigger GFAP release from astrocytes and reactive astrocytes. One way of observing GFAP release might be possible by analyzing astrocyte-derived exosomes through astrocyte-specific markers. These biological analyses must be backed by global, longitudinal cohort studies with rigorously standardized measurement methods, along with the support of imaging probes for reactive astrocytes. Only with such a thorough and meticulous approach can we move beyond superficial associations and harness GFAP as a precise, reliable biomarker for astrocyte reactiveness in neurodegenerative diseases.

## Data Availability

Not applicable. The study contains publicly available data from published studies.
